# Increased expression of OX40 is associated with progressive disease in patients with HTLV-1-associated myelopathy/tropical spastic paraparesis

**DOI:** 10.1186/1742-4690-10-51

**Published:** 2013-05-07

**Authors:** Mineki Saito, Reiko Tanaka, Shiho Arishima, Toshio Matsuzaki, Satoshi Ishihara, Takashi Tokashiki, Yusuke Ohya, Hiroshi Takashima, Fujio Umehara, Shuji Izumo, Yuetsu Tanaka

**Affiliations:** 1 Department of Immunology, Graduate School of Medicine, University of the Ryukyus, 207 Uehara, Okinawa 903-0215, Japan; 2 Division of Molecular Pathology, Center for Chronic Viral Diseases, Kagoshima University Graduate School of Medical and Dental Sciences, 8-35-1 Sakuragaoka, Kagoshima 890-8520, Japan; 3 Department of Neurology and Geriatrics, Kagoshima University Graduate School of Medical and Dental Sciences, 8-35-1 Sakuragaoka, Kagoshima 890-8520, Japan; 4 Department of Cardiovascular Medicine, Nephrology and Neurology, Graduate School of Medicine, University of the Ryukyus, 207 Uehara, Okinawa 903-0215, Japan; 5 Department of Neurology, Nanpu Hospital, 14-3 Nagata-cho, Kagoshima 892-8512, Japan; 6 Present Address: Department of Microbiology, Kawasaki Medical School, 577 Matsushima, Kurashiki 701-0192, Japan

**Keywords:** HTLV-1, OX40, HAM/TSP, ADCC, Immunotherapy

## Abstract

**Background:**

OX40 is a member of the tumor necrosis factor receptor family that is expressed primarily on activated CD4^+^ T cells and promotes the development of effector and memory T cells. Although OX40 has been reported to be a target gene of human T-cell leukemia virus type-1 (HTLV-1) viral transactivator Tax and is overexpressed *in vivo* in adult T-cell leukemia (ATL) cells, an association between OX40 and HTLV-1-associated inflammatory disorders, such as HTLV-1-associated myelopathy/tropical spastic paraparesis (HAM/TSP), has not yet been established. Moreover, because abrogation of OX40 signals ameliorates chronic inflammation in animal models of autoimmune disease, novel monoclonal antibodies against OX40 may offer a potential treatment for HTLV-1-associated diseases such as ATL and HAM/TSP.

**Results:**

In this study, we showed that OX40 was specifically expressed in CD4^+^ T cells naturally infected with HTLV-1 that have the potential to produce pro-inflammatory cytokines along with Tax expression. We also showed that OX40 was overexpressed in spinal cord infiltrating mononuclear cells in a clinically progressive HAM/TSP patient with a short duration of illness. The levels of the soluble form of OX40 (sOX40) in the cerebrospinal fluid (CSF) from chronic progressive HAM/TSP patients or from patients with other inflammatory neurological diseases (OINDs) were not different. In contrast, sOX40 levels in the CSF of rapidly progressing HAM/TSP patients were higher than those in the CSF from patients with OINDs, and these patients showed higher sOX40 levels in the CSF than in the plasma. When our newly produced monoclonal antibody against OX40 was added to peripheral blood mononuclear cells in culture, HTLV-1-infected T cells were specifically removed by a mechanism that depends on antibody-dependent cellular cytotoxicity.

**Conclusions:**

Our study identified OX40 as a key molecule and biomarker for rapid progression of HAM/TSP. Furthermore, blocking OX40 may have potential in therapeutic intervention for HAM/TSP.

## Background

Human T-cell leukemia virus type 1 (HTLV-1) was the first human oncogenic retrovirus to be identified and associated with distinct human diseases such as adult T-cell leukemia (ATL) [1,2] and HTLV-1-associated myelopathy/tropical spastic paraparesis (HAM/TSP) [3,4]. HAM/TSP is a chronic progressive myelopathy characterized by spastic paraparesis, sphincter dysfunction, and mild sensory disturbance in the lower extremities [5]. In addition to neurological symptoms, some HAM/TSP patients also exhibit autoimmune-like disorders such as uveitis, arthritis, T-lymphocyte alveolitis, polymyositis, and Sjögren syndrome [6]. Major pathological features of HAM/TSP are chronic inflammation of the spinal cord, characterized by perivascular lymphocytic cuffing and parenchymal lymphocytic infiltration that includes HTLV-1-infected CD4^+^ T cells [7]. In HAM/TSP patients, the median HTLV-1 proviral load (PVL), which reflects the *in vivo* number of HTLV-1-infected lymphocytes, is more than 10 times higher than that in asymptomatic carriers (ACs) [8]. An increase in PVL typically coincides with worsening of clinical symptoms [9]. Increased concentrations of inflammatory markers such as neopterin [10], tumor necrosis factor (TNF)-α, interleukin (IL)-6, and interferon (IFN)-γ [11], and increase in HTLV-1 antigen-specific intrathecal antibody synthesis [12] have been observed in the cerebrospinal fluid (CSF) of HAM/TSP patients. More recently, it has been reported that IFN-stimulated genes were overexpressed in circulating leukocytes and the expression correlated with the clinical severity of HAM/TSP [13]. These findings indicate that a pro-inflammatory environment, associated with increased numbers of HTLV-1-infected cells, is a characteristic immunologic profile of HAM/TSP.

OX40, also known as CD134 or TNFRSF4, is a member of the TNF co-stimulatory receptor family and is expressed on activated T cells [14]. OX40 is specifically up-regulated by the HTLV-1 viral transactivator Tax [15,16]. The ligand of OX40 (OX40L), which belongs to the TNF superfamily, was first identified as glycoprotein 34 (gp34) on HTLV-1-transformed cells [17], and it was later found to bind OX40 [18]. OX40-OX40L interactions alter the activity and differentiation of many kinds of immune cells, including regulatory T cells (Tregs), T cells, antigen-presenting cells (APCs), natural killer (NK) cells, and natural killer T (NKT) cells [14]. Previous studies have reported that OX40 is constitutively expressed in ATL cells and participate in cell adhesion [19]. Specifically, OX40 and OX40L directly mediate the adhesion of activated normal CD4^+^ T cells, as well as HTLV-1-transformed T cells, to vascular endothelial cells [20]. Immunohistochemical staining of skin biopsy specimens from ATL patients also showed constitutive expression of OX40, suggesting its role in leukemic cell infiltration, in addition to *in vivo* cell adhesion [19].

Recent research has also shown the importance of OX40-OX40L interactions in the development of immune-mediated diseases. In particular, a strong reduction in disease severity or a complete lack of disease has been reported when OX40 or OX40L is absent or neutralized in animal models of multiple sclerosis (MS) [21], allergic asthma [22], colitis [23], diabetes [24], arthritis [25], atherosclerosis [26], graft versus host disease [27], and allograft rejection [28]. Although HTLV-1 causes an aggressive T cell malignancy (i.e., ATL) and chronic inflammatory diseases such as HAM/TSP, an association of OX40 with the inflammatory diseases observed in HTLV-1-infected individuals has not yet been established.

In this study, we investigated the expression of OX40 in HAM/TSP patients and found that the increased expression of OX40 is associated with the rapidly progressive disease. We also used an in-house monoclonal antibody (mAb) against human OX40 to test the potential of OX40 as a target molecule for immunotherapy.

## Results

### Tax-dependent constitutive expression of OX40 in HTLV-1-infected T cells

**Figure 1:**
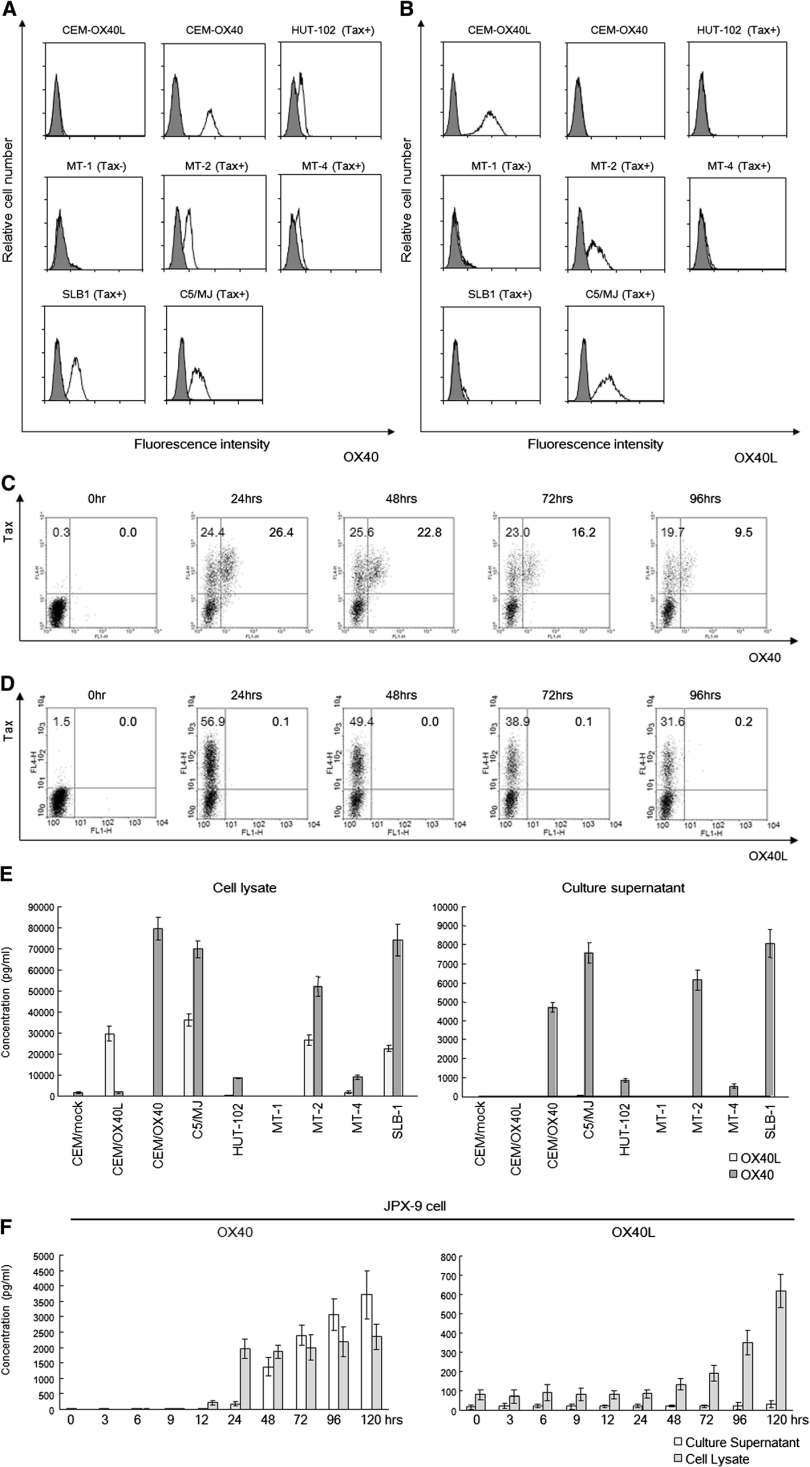
**Tax-dependent constitutive expression of OX40 in HTLV-1-infected T-cell lines and Tax-inducible JPX-9 cell line. A.** Representative histograms of OX40 expression in 6 HTLV-1 infected T-cell lines (HUT-102, MT-1, MT-2, MT-4, C5/MJ, SLB-1) and two HTLV-1-uninfected T-cell lines (CEM-OX40L and CEM-OX40). Shaded histograms represent the isotype control. Tax+ or Tax- means whether these cells express Tax (Tax+) or not (Tax-). **B.** Representative histograms of OX40L expression in 6 HTLV-1 infected T-cell lines (HUT-102, MT-1, MT-2, MT-4, C5/MJ, SLB-1) and two HTLV-1-uninfected T-cell lines (CEM-OX40L and CEM-OX40). Shaded histograms are isotype controls. **C.** Flow cytometric analysis of expression of OX40 after induction of Tax in JPX-9 cells. **D.** Flow cytometric analysis of expression of OX40L after induction of Tax in JPX-9 cells. **E.** Soluble OX40 and OX40L levels in cell culture supernatant and cell lysate from 6 HTLV-1 infected T-cell lines (HUT-102, MT-1, MT-2, MT-4, C5/MJ, SLB-1) and three HTLV-1-uninfected T-cell lines (CEM-mock, CEM-OX40L and CEM-OX40). **F.** Soluble OX40 and OX40L levels in cell culture supernatant and cell lysate from JPX-9 cell line treated with CdCl_2_ along with the induction of viral transactivator Tax.

OX40 and OX40L have been reported to be overexpressed in HTLV-1-infected human T-cells lines [15,19,20]. These findings were obtained using northern blot or western blot analysis using whole cells; hence, our first aim was to confirm and extend these findings at the single-cell level using flow cytometry. Therefore, we used mAbs against human OX40 (clone B-7B5) and human OX40L (clone 5A8) produced in our laboratory. We analyzed six HTLV-1-infected human T-cell lines (HUT-102, MT-1, MT-2, MT-4, SLB-1, and C5/MJ). C5/MJ, SLB-1, and MT-4 cells have not been previously tested for OX40/OX40L expression. As shown in Figure 1A, expression levels were different in each cell line: OX40 was overexpressed on the surface of the Tax positive (Tax+) T-cell lines (HUT-102, MT-2, MT-4, SLB-1, and C5/MJ), but OX40 was not expressed on the surface of the Tax negative (Tax-) MT-1 cell line or the uninfected T cell line (CEM-OX40L). Consistent with previous studies, these findings suggested that OX40 expression is Tax dependent. In contrast, OX40L was not always expressed on the surface of HTLV-1-infected human T-cell lines or on the uninfected T cell line (CEM-OX40), irrespective of Tax expression (Figure 1B).

Next, we confirmed whether OX40 and OX40L protein expression on the cell surface is induced by Tax at the single-cell level by flow cytometry. We used JPX-9 cells [29], a Jurkat (HTLV-1 negative human T cell leukemia cell line) subclone generated by stable transfection of a functional Tax expression-plasmid vector, and induced Tax expression by adding CdCl_2_ into the culture medium (final concentration: 10 μM). As shown in Figure 1C, treatment of JPX-9 cells with CdCl_2_ induced expression of Tax, and OX40 was expressed exclusively in cells that also expressed Tax. In contrast, OX40L was not expressed in JPX-9 cells even after 96 hours post Tax-induction (Figure 1D).

Previous reports indicated that the soluble forms of OX40 (sOX40) and OX40L (sOX40L) were detectable in serum of patients with autoimmune disease and cancer [30,31]. We therefore examined whether sOX40 and sOX40L levels were elevated in culture supernatants from HTLV-1 infected T-cell lines and JPX-9 cells before and after induction of Tax. In agreement with our flow cytometry data (Figure 1A), sOX40 was detected in both culture supernatants and cell lysates of Tax positive C5/MJ, HUT102, MT-2, MT-4, and SLB-1 cells (Figure 1E, gray bar). However, sOX40L was not detected in culture supernatants of any of the samples tested, but it was readily detectable in cell lysates of Tax positive C5/MJ, MT-2, MT-4 and SLB1 cells (Figure 1E, light gray bar). We next examined whether soluble OX40 and OX40L are induced by Tax in JPX-9 cells. Addition of CdCl_2_ to the culture medium of JPX-9 cells resulted in a concomitant increase in sOX40 expression within 24 hours, indicating a strong correlation and functional link between Tax and sOX40 expression (Figure 1F, left panel). Interestingly, although OX40L was already present before induction of Tax, OX40L expression was increased after 24 hours but was never released into the culture supernatant as sOX40L within 120 hours after induction of Tax (Figure 1F, right panel).

### Functional OX40 is specifically expressed on the surface of T cells naturally infected with HTLV-1 that have the potential to produce pro-inflammatory cytokines

**Figure 2:**
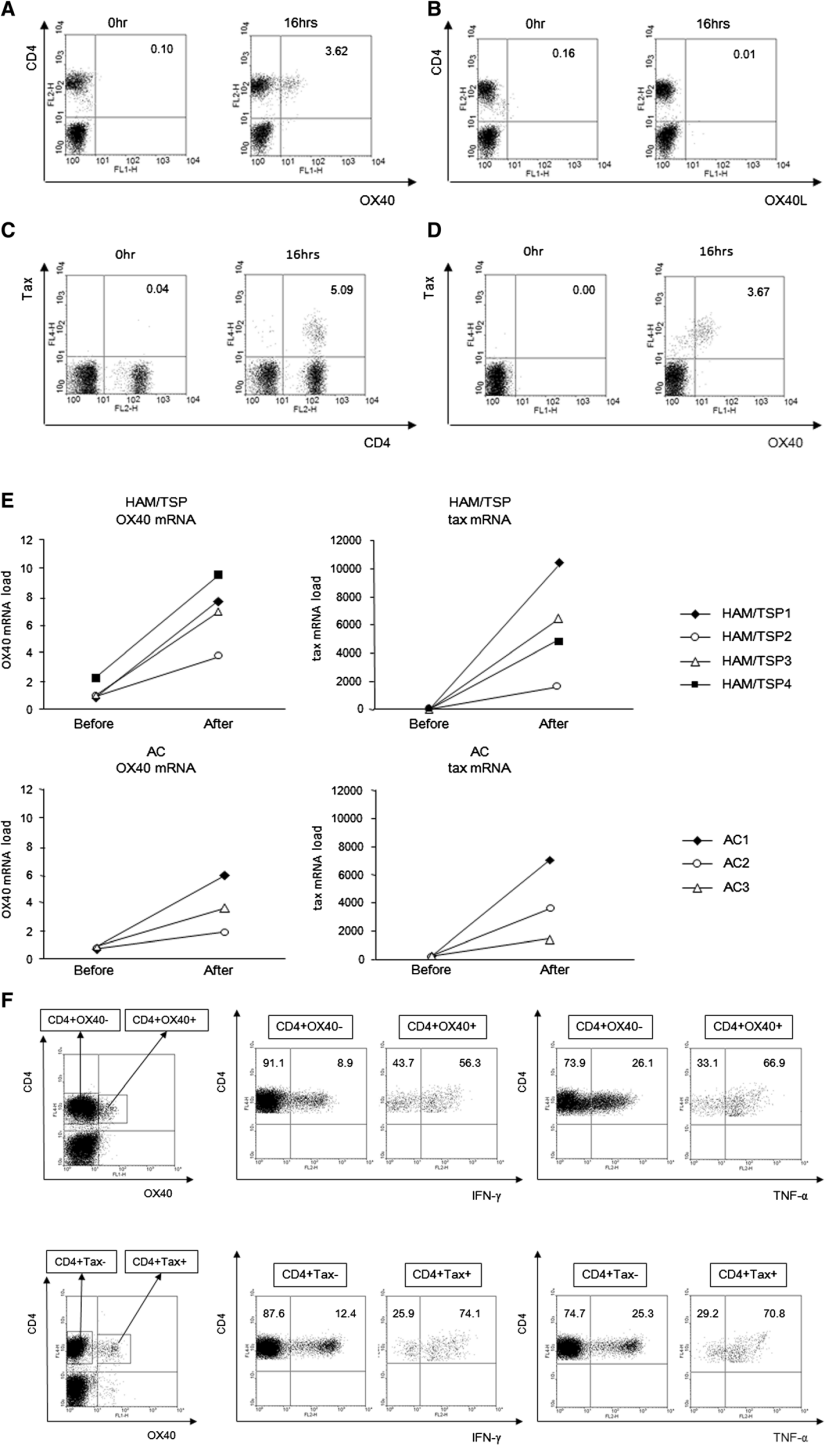
**OX40 is specifically expressed on the surface of T cells naturally infected with HTLV-1 that have the potential to produce pro-inflammatory cytokines. A.** OX40 was detected on CD4^+^ T cells of HAM/TSP patient with anti-OX40 mAb (clones B-7B5) after 16 hours *in vitro* cultivation in the absence of any growth factors or mitogen. **B.** OX40L was not detected on CD4^+^ T cells of HAM/TSP patient with anti-OX40L mAb (clones 5A8) after 16 hours *in vitro* cultivation in the absence of any growth factors or mitogen. **C.** Tax protein was detected in CD4^+^ T cells of HAM/TSP patient after 16 hours *in vitro* cultivation. **D.** OX40 was expressed almost exclusively in naturally infected CD4^+^ T cells that also expressed Tax in HAM/TSP patient. **E.** Both HTLV-1 tax and OX40 mRNA expression in CD4^+^ T cells was increased after 16 hours *in vitro* cultivation. **F.** The frequency of pro-inflammatory cytokine positive cells within the OX40^+^CD4^+^ and Tax^+^CD4^+^ populations from HTLV-1 infected individuals are significantly higher than OX40^-^CD4^+^ and Tax^-^CD4^+^ T cells, respectively (p<0.001, Student’s t- test). One representative experiment of HAM/TSP patient (HAM/TSP1) is shown.

Next, we tested whether OX40 or OX40L expression is also activated in naturally infected T cells isolated directly from HTLV-1-infected individuals. PBMCs were collected from three non-infected controls (NCs), three ACs, and four HAM/TSP patients. PBMCs were isolated from blood samples and harvested directly, or after a 16-hour in vitro cultivation in the absence of any growth factors or mitogens. After harvesting, cell samples were fixed and processed for concomitant detection of Tax, OX40, or OX40L, and CD4 expression by flow cytometry. Similar to the findings for JPX-9 cells, OX40 was detected with an anti-OX40 mAb (clones B-7B5) after 16 hours of in vitro cultivation (Figure 2A), but OX40L was not detected in cultured PBMCs from a HAM/TSP patient (HAM/TSP1) (Figure 2B). Figure 2C shows that the Tax protein was detected in CD4^+^ T cells after cultivation. Similar to the JPX-9 cell experiments, OX40 was expressed almost exclusively in the naturally infected CD4^+^ T cells that also expressed Tax (Figure 2D). Similar findings were observed in all samples tested, irrespective of disease status (i.e., HAM/TSP or ACs) (Additional file 1: Figure S1 and Additional file 2: Table S1). The cells from NCs did not express either OX40 or Tax in CD4^+^ T cells, before or after cultivation (data not shown). Real time RT-PCR also showed that mRNA expression of HTLV-1 tax and OX40 in CD4^+^ T cells was increased after cultivation, both in HAM/TSP patients and ACs (Figure 2E).

It has recently been reported [32], that the expression of another co-stimulatory member of the TNFR family, 4-1BB, is also up-regulated ex vivo in CD4^+^ T cells from HTLV-1-infected individuals, and it was found to be correlated with Tax expression (Additional file 1: Figure S2A and B). However, the expression of OX40 is more specific for Tax^+^CD4^+^ cells than 4-1BB (Figure 2D and Additional file 1: Figure S2C).

Next, we sought to determine if OX40, expressed on the surface of Tax^+^CD4^+^ T cells from HTLV-1-infected individuals, is functional. We incubated aliquots of Fc-blocked PBMCs with biotinylated recombinant soluble OX40L at a concentration of 2.5 mg/ml for 30 min on ice. Cells were then fixed and processed for concomitant detection of Tax, CD4, and PE-streptavidin by flow cytometry. As shown in Additional file 1: Figure S3, the frequency of CD4^+^ T cells that were positively stained with biotinylated recombinant soluble OX40L and PE-streptavidin was similar to the percentage of CD4^+^ T cells stained by anti-OX40 mAb, indicating that these cells expressed functional OX40.

**Figure 3:**
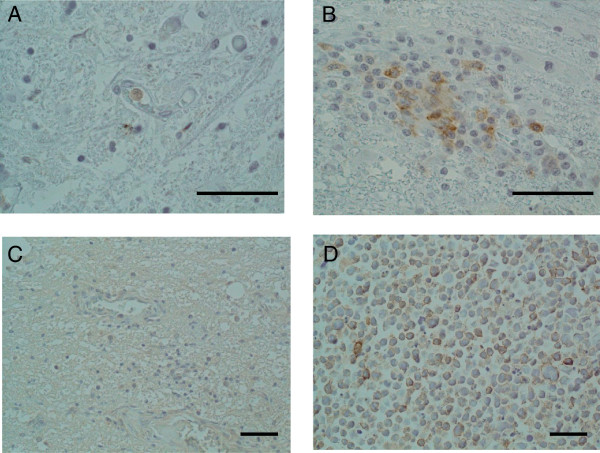
**Increased expression of OX40 in vivo in rapidly progressive HAM/TSP patients.A.** The plasma levels of soluble OX40 (sOX40) measured by ELISA. The plasma levels of sOX40 in typical HAM/TSP patients (chronic HAM: n=20), asymptomatic carriers (ACs: n=9) and normal uninfected healthy controls (NCs: n=13). **B.** No correlation between the plasma levels of sOX40 and HTLV-1 proviral load (tax copies/10,000PBMCs) from 29 HTLV-1 infected individuals (20 chronic HAM/TSP patients and 9 ACs). Data were analyzed by Spearman rank correlation. **C.** The cerebrospinal fluid (CSF) levels of sOX40 in rapidly progressive HAM/TSP patients (n=3), chronic HAM/TSP patients (n=22) and other neurological diseases including multiple sclerosis (MS) (n=12), aseptic meningitis (n=8), systemic lupus erythematosus (SLE) with neurological manifestations (n=5), chronic inflammatory demyelinating polyneuropathy (CIDP) (n=9), Guillain-Barré syndrome (GBS) (n=6), and amyotrophic lateral sclerosis (ALS) (n=9). Chronic HAM/TSP means typical cases fulfilling diagnostic criteria and rapidly progressive HAM/TSP is defined by patients’ incapacity to walk unaided within three months after symptoms’ onset. **D.** The levels of sOX40 in the CSF from HTLV-1 infected other inflammatory neurological diseases (HTLV-1+ OINDs), i.e. any inflammatory neurological disorders except for HAM/TSP which occurred in HTLV-1 infected individuals, was not significantly different from that of chronic HAM/TSP, whereas the levels of sOX40 from HTLV-1+ OINDs was significantly increased than that of non-infected OINDs (HTLV-1- OINDs). HTLV-1+ OINDs: 1 multiple sclerosis (MS), 1 SLE with neurological manifestations, 4 aseptic meningitis. HTLV-1- OINDs: 9 MS, 5 SLE with neurological manifestations, 7 CIDP, 5 GBS.

We further analyzed if CD4^+^OX40^+^ T cells in HAM/TSP patients were capable of producing the inflammatory and neurotoxic cytokines, IFN-γ and TNF-α, which, according to the bystander damage hypothesis, could cause central nervous system (CNS) inflammation and demyelination seen in HAM/TSP patients [33,34]. The frequency of pro-inflammatory cytokine positive cells within the OX40^+^CD4^+^ and Tax^+^CD4^+^ populations from HAM/TSP patients are significantly higher than OX40^-^CD4^+^ and Tax^-^CD4^+^ T cells, respectively (p < 0.001, Student's *t*- test) (Figure 2F and Table 1).

**Table 1:** The expression of pro-inflammatory cytokines in peripheral blood mononuclear cells of HTLV-1 infected individuals

**Case**	**Age**	**Sex**	**PVL**^**a**^	**%IFN-**γ^**+**^**in**	**%IFN-**γ^**+**^**in**	**%IFN-**γ^**+**^**in**	**%IFN-**γ^**+**^**in**	**% TNF-α**^**+**^**in**	**% TNF-α**^**+**^**in**	**% TNF-α**^**+**^**in**	**% TNF-α**^**+**^**in**
				**CD4**^**+**^**OX40**^**+b**^	**CD4**^**+**^**OX40**^**-**^	**CD4**^**+**^**Tax**^**+c**^	**CD4**^**+**^**Tax**^**-**^	**CD4**^**+**^**OX40**^**+**^	**CD4**^**+**^**OX40**^**-**^	**CD4**^**+**^**Tax**^**+**^	**CD4**^**+**^**Tax**^**-**^
HAM/TSP7	68	F	1200	56.3	8.9	74.1	12.4	66.9	26.1	70.8	25.3
HAM/TSP8	68	F	1118	77.2	5.0	91.5	4.7	84.1	10.0	87.6	10.7
HAM/TSP9	71	F	1424	64.8	4.7	80.1	5.3	70.4	18.8	80.5	16.6
mean±SE	69.0±1.0		1247±65	66.1±4.3	6.2±1.0	81.9±3.6	7.5±1.7	73.8±3.7	18.3±3.3	79.6±3.4	17.5±3.0
AC4	74	F	435	61.9	13.8	61.8	13.6	30.8	11.5	25.0	11.1
AC5	76	M	139	55.3	24.9	43.0	27.8	38.3	22.1	47.9	14.3
AC6	71	F	250	47.3	15.0	62.1	34.6	15.8	10.5	34.8	21.9
mean±SE	73.7±1.5		275±61	54.8±3.0	17.9±2.5	55.6±4.5	25.3±4.4	28.3±4.7	14.7±2.6	35.9±4.7	15.8±2.3

HAM/TSP: HTLV-1 associated myelopathy/tropical spastic paraparesis. AC: asymptomatic carrier. PVL: Proviral load.

^a^ PVL: HTLV-1 tax copy number per 10^4^ peripheral blood mononuclear cells (PBMCs).

^b “^%IFN-γ^+^ in CD4^+^OX40^+”^ means the frequency of IFN-γ^+^ cells in the CD4^+^OX40^+^ cell gate.

^c “^%IFN-γ^+^ in CD4^+^Tax^+”^ means the frequency of IFN-γ ^+^ cells in the CD4^+^Tax^+^ cell gate.

### Increased expression of OX40 in vivo in rapidly progressive HAM/TSP patients

To investigate if OX40 expression is associated with in vivo pathogenesis of HAM/TSP, we first measured the plasma concentration of sOX40 and sOX40L in 20 chronic HAM/TSP patients, 9 ACs, and 13 NCs by ELISA by using monoclonal antibodies generated in our laboratory (Figure 3A). None of the samples had detectable levels of sOX40L (data not shown), but we could readily detect sOX40. The median level of sOX40 in NCs was 149.5 pg/ml (range 13–328 pg/ml). Significantly higher sOX40 levels were found in chronic HAM/TSP patients (median 395.2 pg/ml, range 113–1295 pg/ml) and ACs (median 423.8 pg/ml, range 201–881 pg/ml) than in NCs (p=0.0043 for differences between HAM/TSP and NCs, p=0.0020 for differences between ACs and NCs). The difference between chronic HAM/TSP patients and ACs was not statistically significant. No positive correlation was found between sOX40 in the plasma and HTLV-1 PVL in infected individuals (i.e., chronic HAM/TSP patients and ACs) (Spearman’s rank correlation coefficient n=29, r=0.031, P=0.873; Figure 3B). We then tested disease specificity by measuring the levels of sOX40 in the CSF from both rapidly progressive and chronic HAM/TSP patients, and in patients with other neurological disorders, with and without inflammation (e.g., 12 MS, 8 aseptic meningitis, 5 systemic lupus erythematosus with neurological manifestations, 9 chronic inflammatory demyelinating polyneuropathy, 6 Guillain-Barré syndrome, and 9 amyotrophic lateral sclerosis patients). As shown in Figure 3C, CSF sOX40 levels were markedly increased in patients with rapidly progressive HAM/TSP (n=3) and aseptic meningitis (n=8). The CSF sOX40 levels in other HTLV-1-infected inflammatory neurological diseases, i.e. any inflammatory neurological disorders except for HAM/TSP that occurred in HTLV-1 infected individuals, (HTLV-1+ OINDs, n=6) was not significantly different from chronic HAM/TSP (n=20), whereas the sOX40 level of HTLV-1+ OINDs was significantly increased compared to non-infected OINDs (HTLV-1- OINDs, n=26; Figure 3D).

Of the HAM/TSP patients studied, paired CSF and plasma samples, i.e., blood and CSF were collected on the same day, were available for six patients. HAM/TSP patients No.10-12 had a lower concentration of sOX40 in the CSF than in the plasma (Table 2), and the patients showed a typical clinical course of HAM/TSP (i.e. slowly progressive symmetrical myelopathy) and had no history of rapid exacerbation. In contrast, HAM/TSP patients No.13-15, who had higher concentrations of sOX40 in the CSF than in the plasma, showed a rapidly progressive clinical course (i.e. patients became unable to walk within three months after onset of initial symptoms).

**Table 2:** Clinical and laboratory findings of HAM/TSP patients for whom paired CSF and plasma samples were tested for soluble OX40 (sOX40)

**Case**	**Age**	**Sex**	**Disease**	**HTLV-1 proviral load**	**HTLV-1**	OMDS*	**sOX40**	**sOX40**
			**Duration**	**(copies/10**^**4**^**PBMCs)**	**Ab titer (PA)**		**(Plasma)**	**(CSF)**
HAM/TSP10	67	F	6 years	698	×4096	7	534.9	52.1
HAM/TSP11	29	F	1 year	1138	×16384	2	394.0	54.1
HAM/TSP12	41	F	5 years	800	×16384	4	1459.0	55.6
HAM/TSP13	62	F	1 month	224	×8192	10	626.6	752.1
HAM/TSP14	75	F	3 months	437	×4096	9	337.6	897.4
HAM/TSP15	66	F	2 months	534	×4096	9	423.5	652.5

*OMDS: Osame Motor Disability Score that graded the motor dysfunction from zero (normal walking and running) to 13 (complete bedridden): 1=normal gait but runs slow; 2=abnormal gait; 3=abnormal gait and unable to run; 4=need support while using stairs; 5=need one hand support in walking; 6=need two hands support in walking; 7=need two hands support in walking but is limited to 10 m; 8=need two hands support in walking but is limited to 5 m; 9=unable to walk but able to crawl on hands and knees; 10=crawls with hands; 11=unable to crawl but can turn sideways in bed; 12=unable to turn sideways but can move the toes.

### Expression of OX40 in inflammatory mononuclear cells in spinal cord lesions of HAM/TSP patient with short disease duration and progressive symptoms

**Figure 4:**
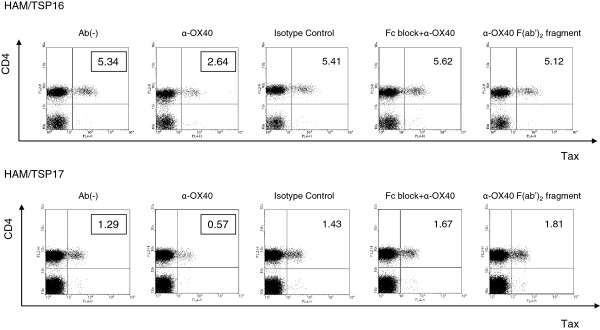
**Expression of OX40 in inflammatory mononuclear cells in spinal cord lesions of HAM/TSP patient with short disease duration and progressive symptoms.** We studied autopsy specimens from 9 HAM/TSP patients by immunohistochemical staining. **A.** No OX40 positive cells are detected in the spinal cord lesion without active inflammation of a HAM/TSP patient with a long duration of illness. Magnification: ×40. **B.** Many infiltrating mononuclear cells are positively stained by anti-OX40 mAb in the spinal cord lesion with active inflammation of HAM/TSP patient with 2.5 years of illness. Magnification: ×40. **C.** There was reduced or no OX40L protein expression in spinal cord tissues of HAM/TSP patients. OX40L showed only low background staining and there was no OX40L positive staining on inflammatory mononuclear cells in the spinal cord lesions. Magnification: ×20. **D.** Positive control staining for OX40L positive CEM-OX40L cells. Magnification: ×20. Bar: 50 μm.

We also examined autopsy specimens from HAM/TSP patients by immunohistochemical staining. Although there was reduced or no OX40 protein expression in HAM/TSP patients who had a long duration of illness and who no longer had active inflammation (a representative example is shown in Figure 4A), we observed marked OX40 expression in inflammatory round-shaped mononuclear cells around the blood vessels in spinal cord lesions from one HAM/TSP patient (Figure 4B). This patient (patient 1 in refs [35-38], who had a shorter disease duration of up to 2.5 years after the onset of neurological symptoms) showed predominant infiltration of CD4^+^ T cells [36] that also expressed tax mRNA [38], pro-inflammatory cytokines [37], and matrix metalloproteinases [39]. In contrast, we observed only low background staining for OX40L in spinal cord tissues of all the HAM/TSP patients examined (a representative example is shown in Figure 4C) compared to positive control (Figure 4D).

### Anti-OX40 monoclonal antibody specifically eliminated naturally infected CD4^+^ T cells via antibody-dependent cell-mediated cytotoxicity (ADCC) in cultured PBMCs

**Figure 5:**
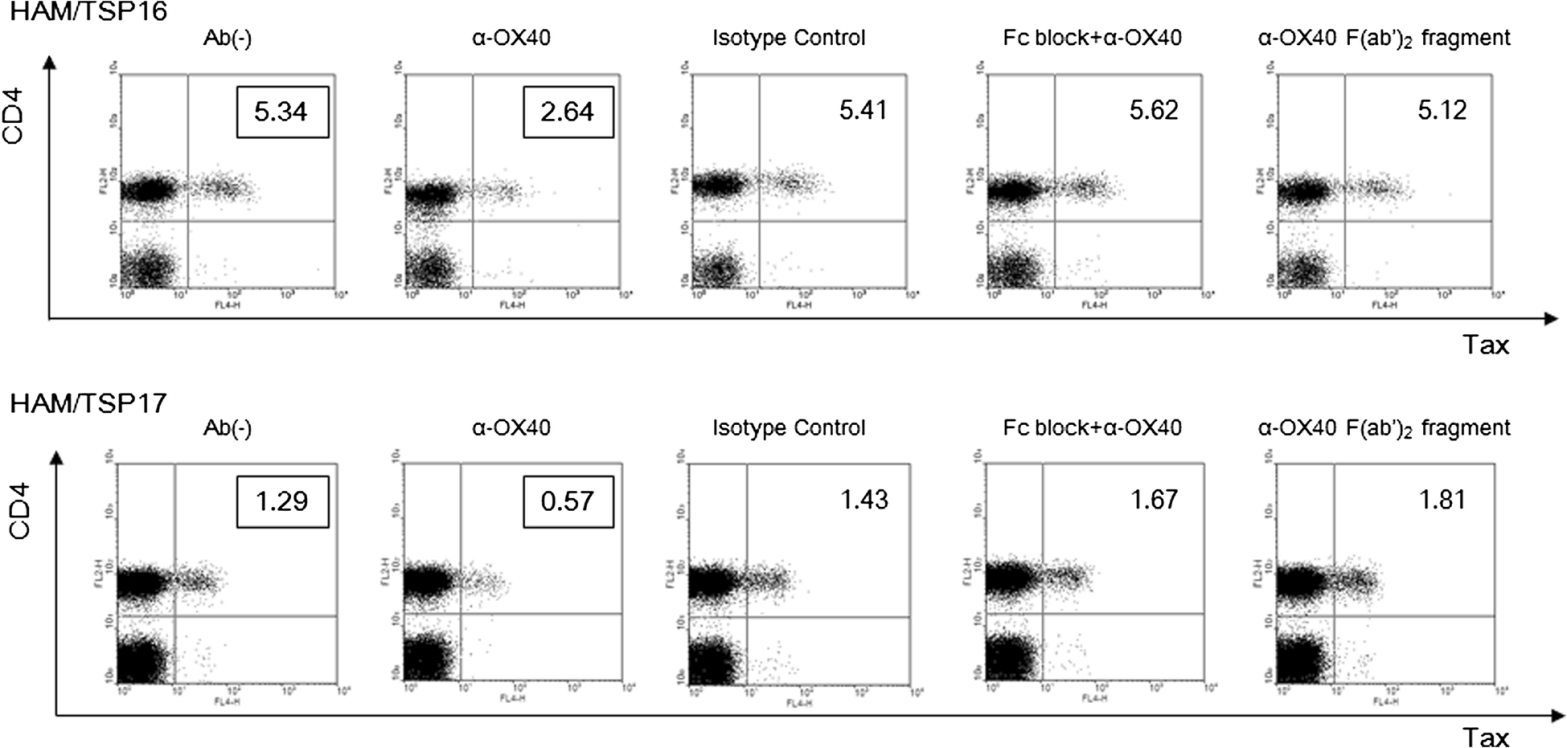
**Anti-OX40 monoclonal antibody specifically eliminated naturally infected CD4**^**+ **^**T cells via antibody-dependent cell-mediated cytotoxicity (ADCC) in cultured PBMCs.** Anti-OX40 mAb (clone B-7B5) reduces the percentage of Tax-positive cells, whereas the isotype control mAb (clone 2C2: anti-HIV-1 gp21, mouse IgG1) has no effect on Tax expression (1st, 2nd, and 3rd panels from left). Culture of PBMCs with anti-CD16/CD32 (Fc receptor) antibody to block Fc receptors abolishes Tax suppression by anti-OX40 mAb (4th panels from left). F(ab’)_2_ fragment do not suppress Tax exppression (right panels).

We investigated the role of OX40 in HTLV-1 naturally infected CD4^+^ T cells, by testing the effects of an anti-human OX40 mAb on Tax expression. As shown in Figure 5, anti-OX40 mAb (clone B-7B5) reduced the percentage of Tax-positive cells, whereas the isotype control mAb (clone 2C2: anti-HIV-1 gp21, mouse IgG1) had no effect on Tax expression (Figure 5, 1st, 2nd, and 3rd panels from left). Culture of PBMCs with anti-CD16/CD32 (Fc receptor) antibody to block Fc receptors abolished Tax suppression by anti-OX40 mAb (Figure 5, 4th panels from left), suggesting that the effect of the anti-OX40 mAb (B-7B5) is mainly mediated by ADCC. We further tested the effects of the F(ab’)_2_ fragment of anti-OX40 mAb (B-7B5) and found that the F(ab’)_2_ fragment did not suppress Tax expression; this finding supports an ADCC mechanism of action of the anti-OX40 mAb (Figure 5, right panels).

### Anti-OX40 monoclonal antibody specifically eliminated OX40-positive HTLV-1 infected cells in cultured PBMCs

**Figure 6:**
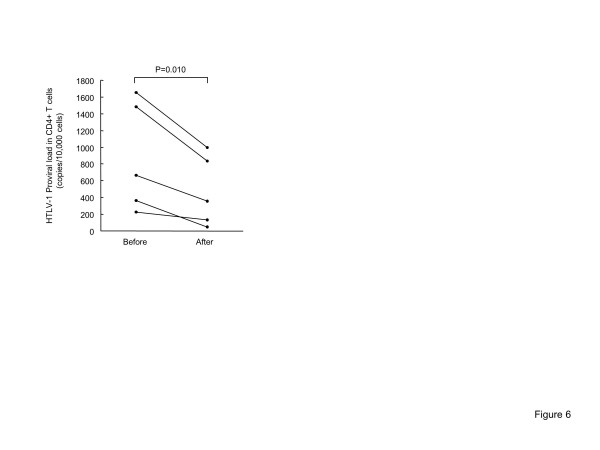
**The HTLV-1 proviral load in CD4**^**+ **^**T cells were significantly reduced after cultivation.** CD4^+^ T cells were isolated from PBMCs before and after cultivation, extracted genomic DNA, and measured the HTLV-1 PVL. HTLV-1 PVL in CD4+ T cells was significantly reduced after culture.

We examined whether suppression of OX40 expression either reduced the frequency of Tax-positive cells or selectively eliminated HTLV-1-infected cells by isolating CD4^+^ T cells from PBMCs before and after culture, extracting genomic DNA, and measuring HTLV-1 PVL. HTLV-1 PVL in CD4^+^ T cells was significantly reduced after culture, suggesting that the anti-OX40 mAb (B-7B5) did not suppress expression of Tax but specifically eliminated OX40-positive HTLV-1 infected cells (Figure 6).

## Discussion

Retroviral infection is characterized by chronic immune-system activation and pro-inflammatory cytokine production [40]. HTLV-1 infection is associated with the development of several different inflammatory conditions, including chronic arthritis, pulmonary alveolitis, polymyositis, Sjögren syndrome, and uveitis [41]. The main pathological feature of HAM/TSP is chronic inflammation of the spinal cord, characterized by perivascular cuffing of mononuclear cells accompanied by parenchymal lymphocytic infiltration. Increased spontaneous peripheral blood lymphocyte proliferation with the production of TNF-α and IFN-γ [42,43], high prevalence of autoantibodies, hypergammaglobulinemia, and complement fixing immune complexes have also been reported in HAM/TSP patients [6]. Recent research has shown the importance of OX40-OX40L interactions in the development of immune-mediated diseases. Specifically, a strong reduction in disease severity, or a complete lack of disease, has been reported when OX40 or OX40L is absent or neutralized in animal models. We therefore hypothesized that the OX40-positive subpopulations of chronically activated T cells exist in naturally HTLV-1-infected cells of HAM/TSP patients. These cells may function to accelerate inflammation, and blocking OX40 may have therapeutic potential in the treatment of HAM/TSP.

Previous reports indicated that OX40 is strongly stimulated by the HTLV-1 viral transactivator Tax [15,19,20]. However, these previous findings were obtained by northern blot or western blot analysis using whole cells. Thus, it was not clear if this induction occurs in naturally infected CD4^+^ T cells of HTLV-1 infected individuals. In the present study, our flow cytometry analysis clearly showed that almost all OX40-positive cells are Tax-positive after short-term culture of naturally HTLV-1-infected cells, suggesting that OX40 is driven exclusively by Tax at the single cell level. In contrast, flow cytometry analysis of JPX-9 cells showed higher percentages of OX40^+^Tax^-^ cells, as well as OX40^+^Tax^+^ cells, after induction of Tax. Although the reasons for this discrepancy are not clear, it can be caused by differential modulation of surface and intracellular protein expression in JPX-9 cells. Our ELISA analysis indicates the existence of intracellular pools of OX40, suggesting that Tax^+^OX40^-^ cells also contain Tax-induced OX40 within JPX-9 cells. While the expression of another co-stimulatory member of the TNFR family, 4-1BB, has also been reported [32], our data indicate that the expression of OX40 was more specific than the expression of 4-1BB in Tax^+^CD4^+^ T cells naturally infected with HTLV-1. It has been previously reported that Tax strongly activates the 4-1BB promoter via a single NF-κB site [32] and the OX40 promoter via 2 NF-κB sites [16]; hence, sustained activation of NF-κB leads to increased expression of numerous pro-inflammatory cytokines and growth factors [44] via NF-κB signaling pathways and ultimately leads to chronic inflammation. In support of these observations, our results show that the frequencies of pro-inflammatory cytokine positive cells within the OX40^+^CD4^+^ and Tax^+^CD4^+^ populations from HAM/TSP patients are significantly higher than OX40^-^CD4^+^ and Tax^-^CD4^+^ T cells, respectively. These cells may be more likely to cross the blood brain barrier and enter the CNS, attract other cells including pro-inflammatory virus-specific CD8^+^ cells, and result in bystander damage to the CNS tissue.

The experimental autoimmune encephalomyelitis (EAE) rat model of human MS shows a selective upregulation of the OX40 protein in encephalitogenic myelin basic protein-specific T cells in the spinal cord during onset of the disease [21]. In contrast, T cells isolated from peripheral blood and spleen of the same animal express low levels of OX40 [21]. This is similar to our present finding, where OX40 was markedly expressed in infiltrating mononuclear cells in spinal cord lesions, but not in uncultivated PBMCs from HAM/TSP patients. Because locally produced pro-inflammatory cytokines up-regulate MHC class II molecules on astrocytes and microglia, increase presentation of CNS antigens, and exert a direct cytotoxic effect on oligodendrocytes [45], the observed expression of OX40 in inflammatory mononuclear cells in spinal cord lesions suggest a role for OX40 in inflammation and neuronal damage that occurs in the CNS of HAM/TSP patients. In the rat EAE model, selective depletion of myelin-reactive T cells, by treatment with an anti-OX40 mAb-conjugated immunotoxin, effectively suppressed disease symptoms [21]. The association of clinical progression of HAM/TSP with increased HTLV-1 PVL in individual patients [9] and the strong stimulation of OX40, together with the expression of the viral transactivator Tax in CD4^+^ T cells, indicates that targeting of OX40 positive T cells by anti-OX40 antibodies may provide a novel therapeutic strategy for the treatment of HAM/TSP.

In the present study, an anti-OX40 monoclonal antibody specifically eliminated naturally infected CD4^+^ T cells in cultured PBMCs via ADCC. This indicates that effector cells may actively lyse HTLV-1-infected CD4^+^ T cells that are bound by the anti-OX40 antibody. Indeed, defucosylated humanized anti-CC chemokine receptor 4 (CCR4) mAbs, which exert a strong ADCC effect, were found to be effective and well tolerated as a treatment for patients with relapsed CCR4-positive ATL or peripheral T-cell lymphoma [46]. In the present study, OX40 expression was not observed in T cells of healthy individuals, and its expression was more specific than CCR4 for HTLV-1-infected cells. This finding suggests that specific elimination of HTLV-1-infected T cells by defucosylated humanized anti-OX40 monoclonal antibodies might be a promising future approach for treatment of HAM/TSP.

We also found that plasma sOX40 levels were more elevated in HTLV-1-infected individuals (chronic HAM/TSP patients and ACs) than in NCs. Three rapidly progressive HAM/TSP patients also showed higher levels of sOX40 in the CSF than in the plasma, suggesting the possibility that sOX40 is released at high levels following strong intrathecal immune activation. In contrast, expression of OX40L was absent in HTLV-1-infected lymphocytes even after short term ex vivo cultivation, in active-chronic spinal cord lesions of HAM/TSP patient, and in plasma of HTLV-1 infected individuals. Therefore, OX40 signals might be generated by interactions with OX40L on antigen presenting cells or endothelial cells at specialized sites such as lymphoid organs. In such lesions, similar to other members of the TNF receptor superfamily like 4-1BB, sOX40 may act as an antagonist to membrane-bound receptors and induce signaling in OX40L^+^ cells to produce cytokines, which in turn drive specific T helper (Th)-cell differentiation and suppress the generation of adaptive Tregs to participate in HAM/TSP pathogenesis.

In conclusion, we demonstrate that OX40 was specifically expressed in CD4^+^ T cells naturally infected with HTLV-1. These cells have the potential to produce pro-inflammatory cytokines along with the expression of the viral transactivator Tax. Higher levels of sOX40 were found in the CSF than in the plasma of three rapidly progressive HAM/TSP patients, and OX40 was overexpressed in the spinal cord infiltrating mononuclear cells of HAM/TSP patient with active disease. Anti-OX40 mAb was able to specifically eliminate HTLV-1-infected CD4^+^OX40^+^Tax^+^ T cells via ADCC. These findings indicate that, in addition to its established role in the regulation of T cell division and survival, OX40 may be a key molecule in the pathogenesis of HAM/TSP, as well as a potential target for immunotherapy.

## Methods

### Patients

Peripheral blood was studied from 23 patients with a clinical diagnosis of HAM/TSP, 9 ACs and 13 uninfected normal controls (NCs). The diagnosis of HAM/TSP was made according to the World Health Organization diagnostic criteria [47]. In this paper, chronic HAM/TSP means typical cases fulfilling diagnostic criteria and rapidly progressive HAM/TSP is defined by patients’ incapacity to walk unaided within three months after symptoms’ onset. This study was approved by the Institutional Review Board of the University of the Ryukyus with license number H21-1-9. All patients provided written informed consent for the collection of samples and subsequent analysis. The CSF and plasma samples were collected before starting therapy. Control subjects of other neurological diseases were MS (n=12), aseptic meningitis (n=8), systemic lupus erythematosus (SLE) with neurological manifestations (n=5), chronic inflammatory demyelinating polyneuropathy (CIDP) (n=9), Guillain-Barré syndrome (GBS) (n=6), and amyotrophic lateral sclerosis (ALS) (n=9). The specimens were stored at −80°C until use.

### Cell culture

Six HTLV-1 infected T-cell lines (HUT-102, MT-1, MT-2, MT-4, SLB-1, C5/MJ) and two HTLV-1-uninfected T-cell lines (CEM-OX40L, CEM-OX40) were used in this study. CEM-OX40 and CEM-OX40L cell lines are stable CEM-derived cell lines expressing the human OX40 or OX40L, respectively. The Tax-inducible JPX-9 cell line is a derivative of the Jurkat HTLV-1 negative human T cell leukemia cell line, which expresses biologically active Tax protein under the control of the metallothionein promoter [29]. These cells were cultured in RPMI 1640 medium supplemented with 10% heat inactivated fetal calf serum (FCS), 50 U/ml penicillin, and 50 μg/ml streptomycin (Wako) at 37°C in 5% CO_2_.

### Preparation of PBMC samples

Fresh peripheral blood mononuclear cells (PBMCs) were isolated on a Histopaque-1077 (Sigma) density gradient centrifugation, washed twice in RPMI 1640 with 10% heat inactivated FCS, and stored in liquid nitrogen as stocked lymphocytes until use. CD4^+^ T cells were isolated from PBMCs by positive immunoselection with the Dynal® CD4-positive isolation kit (Invitrogen), according to the manufacturer’s protocol. In brief, PBMCs were incubated with anti-CD4-coated beads for 30 min at 4°C under gentle tilt rotation. Captured CD4^+^ cells were collected with a magnet (Dynal MPC-S) and detached from beads with DETACHaBEAD CD4/CD8® (Invitrogen). Purity was >99% CD4^+^ T cells, as determined by flow cytometry (data not shown). To induce cytokine production by OX40^+^CD4^+^ T cells, PBMCs were cultivated for 12 hours, then 0.1 ng/ml phorbol myristate acetate (PMA) (Sigma) and 0.5 μg/ml A23187 (Sigma) and 2 mM monensin (Sigma) were added to the culture medium and further cultivated for 5 hours.

### Monoclonal antibodies and reagents

We produced the following monoclonal antibodies (mAbs) in our laboratory: mouse IgG1 mAbs anti-human OX40L (clones 5A8, 8F4), anti-human OX40 (clones B-7B5 and 17D8), anti-HIV-1 p24 (clone 2C2 and NP24), and mouse IgG3 mAb anti-HTLV-1 Tax (clone Lt-4) [48] as well as rat IgG2b mAbs anti-human OX40 (clone W4-54), anti-human OX40L (clone W18) and isotype control anti-HCV (clone MO-8). Some of these mAbs were labeled using FITC, Cy5, or HRP using commercial labeling kits (Dojin or Amersham, Japan) according to the manufacturers’ instructions. Biotinylated recombinant soluble human OX40L (sOX40L in a form of murine CD8-fusion protein) was purchased from Ancell (Bayport, MN) and used with PE-streptavidin (Biolegend) for staining. Recombinant human OX40 ligand/TNFSF4 and recombinant human OX40/TNFRSF4/Fc Chimera were purchased from R&D Systems (Minneapolis, MN) and used for the standard curve in sOX40L and sOX40 ELISA, respectively.

### Immunohistochemistry

Immunohistochemical staining of the spinal cord specimens from HAM/TSP patients was performed on buffered formalin-fixed paraffin-embedded sections using EnVision (DAKO) method for signal detection as described previously [36]. The clinical and pathological characteristics of the patients are described elsewhere [36-39]. The monoclonal antibodies to OX40 (clone B-7B5) and OX40L (clone 8F4) were used at a final concentration of 1 μg/ml.

### Flow cytometry

#### Cell surface staining

After thawing, cells were washed three times with phosphate-buffered saline (PBS) and fixed in PBS containing 2% paraformaldehyde (Sigma) for 20 minutes at 4°C. Fixed cells were washed with PBS containing 7% of normal goat serum (Sigma) and then incubated for 15 minutes at room temperature with various combinations of fluorescence-conjugated mAbs as follows: phycoerythrin-cyanin 5.1 (PC5)-labeled anti-CD4 (13B8.2), PC5-labeled anti-CD8 (B9.11), phycoerythrin (PE)-labeled anti-CD4 (13B8.2) (Beckman Coulter), PE-labeled anti-4-1BB (4B4) (eBioscience), fluorescein isothiocyanate (FITC)-labeled anti-OX40 (B-7B5) and OX40L (5A8). Isotype matched mouse immunoglobulins were used as a control. After the staining procedure, the cells were washed twice and analyzed by standard flow cytometry using a FACS Calibur and Cell Quest software (BD).

#### Concomitant detection of intracellular and cell surface molecules

For intracellular staining of Tax and/or cytokines, surface stained cells were washed and permeabilized with PBS/7% normal goat serum containing 0.2% saponin (Sigma) (PBS-SAPO) for 10 minutes at room temperature. Permeabilized cells were then washed twice and resuspended in PBS-SAPO containing FITC or cyanin 5 (Cy5)-labeled anti-Tax mAb (Lt-4), PE-labeled anti TNF-α (BD Pharmingen) or PE-labeled IFN-γ (BD Pharmingen) mAb for 20 minutes at room temperature. Finally, the cells were washed twice and analyzed by flow cytometry.

### Flow cytometry based binding assay

To determine whether cell surface OX40 is functional, aliquots of Fc-blocked cells were incubated with biotinylated recombinant soluble OX40L at a concentration of 2.5 mg/ml for 30 min on ice, followed by staining with PE-streptavidin (Biolegend) for 30 min on ice. After the staining procedure, the cells were washed twice and analyzed by flow cytometry.

#### ELISA

Cell lysates were prepared by lysis of 2 × 10^7^ cells in 1ml of a lysis buffer (10 mM Tris–HCl, pH8.0, 140 mM NaCl, 3 mM MgCls, 2 mM phenylmethylsulfonyl fluoride, 0.5% Nonidet P-40) on ice for 20 min, followed by centrifugation at 13,000 × g for 10 min at 4°C. Both OX40 and OX40L levels in cell lysates, culture supernatants, plasma and CSF were assayed by in house made sandwich ELISA using monoclonal antibodies against OX40 (clone 17D8 for capture and W4-54 for detection) and OX40L (clone 8F4 for capture and W18 for detection). Briefly, 96-well Immuno Module/Strip Plates (Nunc) was coated with either anti-OX40 monoclobal antibody (clone 17D8) or anti-OX40L monoclobal antibody (clone 8F4) at 4°C overnight, then blocked with 1% casein in 0.02% thimerosal-PBS at room temperature for 30 min. After washing plates three times with wash buffer (PBS with 0.05% Tween 20, pH 7.5), 50 μl of irrelevant mouse IgG1 (anti-HIV1 p24 mAb NP24) was added into each well as a blocking antibody. OX40 or OX40L standard was diluted to 4,000 pg/ml in dilution buffer (PBS with 0.1% BSA, 0.5% Triton X100, 0.05% Tween20), and two-fold serial dilutions were performed ranged from 4,000 to 16 pg/ml. Then 50 μl of the diluted standard or samples (cell lysates, culture supernatants, plasma and CSF) were added into 96-well plates and incubated one hour at room temparature. After washing plates three times, 50 μl each of diluted (0.2 μg/μl) anti-OX40 monoclobal antibody (clone W4-54) or anti-OX40L monoclobal antibody (clone W18) conjugated to HRP was added as detection antibody and incubated for one hour at room temperature. Color reactions using alkaline-phosphatase substrate (Sigma-Aldrich) were then evaluated by Model 680 Microplate Reader (Bio-Rad) reading at 450 nm with reference at 630 nm, and the data was analyzed using the Microplate manager III software (Bio-Rad). Results are shown as mean ± SE for duplicate wells. Human interleukin-2 soluble receptor alpha (IL-2sRα) was measured by ELISA according to the manufacturer’s instruction (Quantikine Human IL-2sRα Immunoassay, R&D Systems, Inc. MN).

#### Genomic DNA, RNA extraction and cDNA synthesis

Genomic DNA was extracted from the frozen PBMCs by QIAamp blood kit (QIAGEN, Tokyo, Japan). RNA from 1×10^5^ enriched CD4^+^ T cells was extracted using RNeasy Mini Kit with on-column DNase digestion (QIAGEN, Tokyo, Japan) according to the manufacturer’s instructions. Complementary DNA (cDNA) was synthesized using PrimeScript® RT reagent Kit (Takara, Kyoto, Japan). All reaction procedures were performed as suggested by the manufacturer.

#### Quantification of HTLV-1 proviral load and anti-HTLV-1 antibody titers

To examine the HTLV-1 PVL, we carried out a quantitative PCR method using Thermal Cycler Dice® Real Time System (Takara, Japan) with 100 ng of genomic DNA (roughly equivalent to 10^4^ cells) from PBMCs samples as reported previously [8]. Based on the standard curve created by four known concentrations of template, the concentration of unknown samples were determined. Using β-actin as an internal control, the amount of HTLV-1 proviral DNA was calculated by the following formula: copy number of HTLV-1 tax per 1 × 10^4^PBMCs = [(*copy number of tax*)/(*copy number of β* − *actin*/2)] × 10^4^. All samples were performed in triplicate. Serum HTLV-1 antibody titers were determined by a particle agglutination method (Serodia-HTLV-1®, Fujirebio, Japan).

#### Real-Time RT-PCR analysis

We used the real-time RT-PCR method to carry out a quantitative analysis of the expression of the tax and OX40 mRNA by using Thermal Cycler Dice® Real Time System (Takara, Japan) as reported previously [49]. HTLV-1 tax or OX40 mRNA load was calculated by the following formula: HTLV - 1 tax mRNA load = value of tax/value of HPRT (Hypoxanthine Phosphorisbosytransferase). OX40 mRNA load = value of OX40/value of HPRT. We used aliquots of the same standard MT-2 cDNA preparation for all assays and the correlation values of standard curves were always more than 99%. The sequences of primers for tax mRNA detection were as follows: 5′- ATC CCG TGG AGA CTC CTC AA-3′ and 5′- ATC CCG TGG AGA CTC CTC AA-3′, and the probe that surrounded the splice junction site of tax mRNA was 5′- TCC AAC ACC ATG GCC CAC TTC CC-3′. The sequences of primers for OX40 mRNA detection were as follows: 5′-AAC CAG GCC TGC AAG CCC T-3′ and 5′- GTC CCT GTC CTC ACA GAT T-3′, and the probe that span the junction between exon 4 and 5 was 5′- ACC AAC TGC ACC TTG GCT GGG AAG CA-3′. We used the HPRT primers and probe set (Applied Biosystems) for internal calibration. All assays were performed in triplicate.

#### Statistical analysis

To test for significant differences among the cell populations between three different groups of subjects (HAM/TSP, ACs and NCs), the Kruskal-Wallis test was employed. For multiple comparisons, we used Sheffe’s F to analyze statistical difference. Correlations between variables were examined by Spearman rank correlation analysis. We made paired comparison of changes in HTLV-1 PVL in CD4^+^ T cells before and after PBMCs cultivation by using a paired t-test. The results represent the mean ± SE where applicable. Values of p<0.05 were considered statistically significant.

## Abbreviations

HTLV-1: Human T-cell leukemia virus type-1; ATL: Adult T-cell leukemia; HAM/TSP: HTLV-1-associated myelopathy/tropical spastic paraparesis; ACs: Asymptomatic carriers; NCs: Normal uninfected healthy controls; MS: Multiple sclerosis; CSF: Cerebrospinal fluid; OINDs: Other inflammatory neurological diseases; ADCC: Antibody-dependent cellular cytotoxicity; PVL: Proviral load; CNS: Central nervous system; SLE: Systemic lupus erythematosus; CIDP: Chronic inflammatory demyelinating polyneuropathy; GBS: Guillain-Barré syndrome; ALS: Amyotrophic lateral sclerosis; HPRT: Hypoxanthine phosphoribosyltransferase

## Competing interests

The authors declare that they have no competing interests.

## Authors’ contributions

MS designed and performed the experiments, analyzed the data, and wrote the paper; TM, SI, TT, YO, and HT provided clinical samples and assembled clinical database; RT, SA, FU, and SI performed experiments, analyzed and interpreted data; YT made contribution to the conception and design of the study. All authors read and approved the final manuscript.

## Supplementary Material

Additional file 1: Figure S1 OX40 was expressed on the surface of Tax^+^ CD4^+^ T cells from HTLV-1 infected individuals. OX40 was detected on CD4^+^ T cells of HAM/TSP patients (HAM/TSP3, 4) and AC (AC1) with anti-OX40 mAb (clones B-7B5) after 16 hours in vitro cultivation in the absence of any growth factors or mitogen (center panels). OX40 was expressed almost exclusively in naturally infected CD4^+^ T cells that also expressed Tax (right panels). **FigureS2.** The expression of 4-1BB on CD4^+^ T cells from HAM/TSP patients. **A**. 4-1BB was detected on both CD4^+^ and CD4- T cells of HAM/TSP patients with anti-4-1BB mAb (clone 4B4, eBioscience) after 16 hours in vitro cultivation in the absence of any growth factors or mitogen. **B**. Tax protein was detected in CD4^+^ T cells after 16 hours in vitro cultivation. **C**. The expression of 4-1BB was associated with the expression of Tax. **Figure S3.** Functional OX40 is specifically expressed on the surface of T cells naturally infected with HTLV-1. To determine if cell surface OX40 is functional, flow cytometry based binding assays have been carried out. Aliquots of Fc-blocked cells were incubated with biotinylated recombinant soluble OX40L at a concentration of 2.5 mg/ml for 30 min on ice. Then cells were washed and stained with PE-streptavidin (Biolegend) and PC5-labeled anti-CD4 for 30 min on ice. After washing, the cells were fixed and processed to detect concomitantly Tax (see Methods). The frequency of CD4^+^ T cells that were positively stained with biotinylated recombinant soluble OX40L and PE-streptavidin was similar to the percentage of CD4^+^ T cells stained by anti-OX40 mAb, indicating that these cells expressed functional OX40. Click here for file

Additional file 2: Table S1 Ex vivo frequency of OX40 and Tax positive T cells in peripheral blood mononuclear cells from HTLV-1 infected individuals. Click here for file

## References

[B1] Yoshida M Seiki M Yamaguchi K Takatsuki K Monoclonal integration of human T-cell leukemia provirus in all primary tumors of adult T-cell leukemia suggests causative role of human T-cell leukemia virus in the disease Proc Natl Acad Sci USA 1984 81 2534 2537 10.1073/pnas.81.8.2534 6326131 PMC345097

[B2] Hinuma Y Nagata K Hanaoka M Nakai M Matsumoto T Kinoshita KI Shirakawa S Miyoshi I Adult T-cell leukemia: antigen in an ATL cell line and detection of antibodies to the antigen in human sera Proc Natl Acad Sci USA 1981 78 6476 6480 10.1073/pnas.78.10.6476 7031654 PMC349062

[B3] Osame M Usuku K Izumo S Ijichi N Amitani H Igata A Matsumoto M Tara M HTLV-I associated myelopathy, a new clinical entity Lancet 1986 1 1031 1032 2871307 10.1016/s0140-6736(86)91298-5

[B4] Gessain A Barin F Vernant JC Gout O Maurs L Calender A de The G Antibodies to human T-lymphotropic virus type-I in patients with tropical spastic paraparesis Lancet 1985 2 407 410 2863442 10.1016/s0140-6736(85)92734-5

[B5] Nakagawa M Nakahara K Maruyama Y Kawabata M Higuchi I Kubota H Izumo S Arimura K Osame M Therapeutic trials in 200 patients with HTLV-I-associated myelopathy/ tropical spastic paraparesis J Neurovirol 1996 2 345 355 10.3109/13550289609146899 8912211

[B6] Nakagawa M Izumo S Ijichi S Kubota H Arimura K Kawabata M Osame M HTLV-I-associated myelopathy: analysis of 213 patients based on clinical features and laboratory findings J Neurovirol 1995 1 50 61 10.3109/13550289509111010 9222342

[B7] Izumo S Umehara F Osame M HTLV-I-associated myelopathy Neuropathology 2000 20 Suppl S65 68 11037191 10.1046/j.1440-1789.2000.00320.x

[B8] Nagai M Usuku K Matsumoto W Kodama D Takenouchi N Moritoyo T Hashiguchi S Ichinose M Bangham CR Izumo S Osame M Analysis of HTLV-I proviral load in 202 HAM/TSP patients and 243 asymptomatic HTLV-I carriers: high proviral load strongly predisposes to HAM/TSP J Neurovirol 1998 4 586 593 10.3109/13550289809114225 10065900

[B9] Takenouchi N Yamano Y Usuku K Osame M Izumo S Usefulness of proviral load measurement for monitoring of disease activity in individual patients with human T-lymphotropic virus type I-associated myelopathy/tropical spastic paraparesis J Neurovirol 2003 9 29 35 12587066 10.1080/13550280390173418

[B10] Nomoto M Utatsu Y Soejima Y Osame M Neopterin in cerebrospinal fluid: a useful marker for diagnosis of HTLV-I-associated myelopathy/tropical spastic paraparesis Neurology 1991 41 457 2006023 10.1212/wnl.41.3.457

[B11] Jacobson S Immunopathogenesis of human T cell lymphotropic virus type I-associated neurologic disease J Infect Dis 2002 186 Suppl 2 S187 192 12424696 10.1086/344269

[B12] Kitze B Usuku K Yashiki S Ijichi S Fujiyoshi T Nakamura M Izumo S Osame M Sonoda S Intrathecal humoral immune response in HAM/TSP in relation to HLA haplotype analysis Acta Neurol Scand 1996 94 287 293 10.1111/j.1600-0404.1996.tb07067.x 8937542

[B13] Tattermusch S Skinner JA Chaussabel D Banchereau J Berry MP McNab FW O’Garra A Taylor GP Bangham CR Systems biology approaches reveal a specific interferon-inducible signature in HTLV-1 associated myelopathy PLoS Pathog 2012 8 e1002480 10.1371/journal.ppat.1002480 22291590 PMC3266939

[B14] Croft M Control of immunity by the TNFR-related molecule OX40 (CD134) Annu Rev Immunol 2010 28 57 78 10.1146/annurev-immunol-030409-101243 20307208 PMC2882161

[B15] Higashimura N Takasawa N Tanaka Y Nakamura M Sugamura K Induction of OX40, a receptor of gp34, on T cells by trans-acting transcriptional activator, Tax, of human T-cell leukemia virus type I Jpn J Cancer Res 1996 87 227 231 10.1111/j.1349-7006.1996.tb00210.x 8613423 PMC5921092

[B16] Pankow R Durkop H Latza U Krause H Kunzendorf U Pohl T Bulfone-Paus S The HTLV-I tax protein transcriptionally modulates OX40 antigen expression J Immunol 2000 165 263 270 10861060 10.4049/jimmunol.165.1.263

[B17] Tanaka Y Inoi T Tozawa H Yamamoto N Hinuma Y A glycoprotein antigen detected with new monoclonal antibodies on the surface of human lymphocytes infected with human T-cell leukemia virus type-I (HTLV-I) Int J Cancer 1985 36 549 555 10.1002/ijc.2910360506 2997042

[B18] Baum PR Gayle RB 3rd Ramsdell F Srinivasan S Sorensen RA Watson ML Seldin MF Baker E Sutherland GR Clifford KN Molecular characterization of murine and human OX40/OX40 ligand systems: identification of a human OX40 ligand as the HTLV-1-regulated protein gp34 EMBO J 1994 13 3992 4001 8076595 10.1002/j.1460-2075.1994.tb06715.x PMC395319

[B19] Imura A Hori T Imada K Kawamata S Tanaka Y Imamura S Uchiyama T OX40 expressed on fresh leukemic cells from adult T-cell leukemia patients mediates cell adhesion to vascular endothelial cells: implication for the possible involvement of OX40 in leukemic cell infiltration Blood 1997 89 2951 2958 9108415

[B20] Imura A Hori T Imada K Ishikawa T Tanaka Y Maeda M Imamura S Uchiyama T The human OX40/gp34 system directly mediates adhesion of activated T cells to vascular endothelial cells J Exp Med 1996 183 2185 2195 10.1084/jem.183.5.2185 8642328 PMC2192546

[B21] Weinberg AD Bourdette DN Sullivan TJ Lemon M Wallin JJ Maziarz R Davey M Palida F Godfrey W Engleman E Selective depletion of myelin-reactive T cells with the anti-OX-40 antibody ameliorates autoimmune encephalomyelitis Nat Med 1996 2 183 189 10.1038/nm0296-183 8574963

[B22] Salek-Ardakani S Song J Halteman BS Jember AG Akiba H Yagita H Croft M OX40 (CD134) controls memory T helper 2 cells that drive lung inflammation J Exp Med 2003 198 315 324 10.1084/jem.20021937 12860930 PMC2194076

[B23] Higgins LM McDonald SA Whittle N Crockett N Shields JG MacDonald TT Regulation of T cell activation in vitro and in vivo by targeting the OX40-OX40 ligand interaction: amelioration of ongoing inflammatory bowel disease with an OX40-IgG fusion protein, but not with an OX40 ligand-IgG fusion protein J Immunol 1999 162 486 493 9886424

[B24] Pakala SV Bansal-Pakala P Halteman BS Croft M Prevention of diabetes in NOD mice at a late stage by targeting OX40/OX40 ligand interactions Eur J Immunol 2004 34 3039 3046 10.1002/eji.200425141 15368274

[B25] Yoshioka T Nakajima A Akiba H Ishiwata T Asano G Yoshino S Yagita H Okumura K Contribution of OX40/OX40 ligand interaction to the pathogenesis of rheumatoid arthritis Eur J Immunol 2000 30 2815 2823 10.1002/1521-4141(200010)30:10<2815::AID-IMMU2815>3.0.CO;2-# 11069062

[B26] van Wanrooij EJ van Puijvelde GH de Vos P Yagita H van Berkel TJ Kuiper J Interruption of the Tnfrsf4/Tnfsf4 (OX40/OX40L) pathway attenuates atherogenesis in low-density lipoprotein receptor-deficient mice Arterioscler Thromb Vasc Biol 2007 27 204 210 10.1161/01.ATV.0000251007.07648.81 17068285

[B27] Tsukada N Akiba H Kobata T Aizawa Y Yagita H Okumura K Blockade of CD134 (OX40)-CD134L interaction ameliorates lethal acute graft-versus-host disease in a murine model of allogeneic bone marrow transplantation Blood 2000 95 2434 2439 10733518

[B28] Curry AJ Chikwe J Smith XG Cai M Schwarz H Bradley JA Bolton EM OX40 (CD134) blockade inhibits the co-stimulatory cascade and promotes heart allograft survival Transplantation 2004 78 807 814 10.1097/01.TP.0000131670.99000.54 15385798

[B29] Nagata K Ohtani K Nakamura M Sugamura K Activation of endogenous c-fos proto-oncogene expression by human T-cell leukemia virus type I-encoded p40tax protein in the human T-cell line, Jurkat J Virol 1989 63 3220 3226 2501514 10.1128/jvi.63.8.3220-3226.1989 PMC250891

[B30] Taylor L Schwarz H Identification of a soluble OX40 isoform: development of a specific and quantitative immunoassay J Immunol Methods 2001 255 67 72 10.1016/S0022-1759(01)00424-0 11470287

[B31] Komura K Yoshizaki A Kodera M Iwata Y Ogawa F Shimizu K Wayaku T Yukami T Murata M Hasegawa M Increased serum soluble OX40 in patients with systemic sclerosis J Rheumatol 2008 35 2359 2362 10.3899/jrheum.080120 18843780

[B32] Pichler K Kattan T Gentzsch J Kress AK Taylor GP Bangham CR Grassmann R Strong induction of 4-1BB, a growth and survival promoting costimulatory receptor, in HTLV-1-infected cultured and patients’ T cells by the viral Tax oncoprotein Blood 2008 111 4741 4751 10.1182/blood-2007-10-115220 18276843

[B33] Ijichi S Izumo S Eiraku N Machigashira K Kubota R Nagai M Ikegami N Kashio N Umehara F Maruyama I An autoaggressive process against bystander tissues in HTLV-I-infected individuals: a possible pathomechanism of HAM/TSP Med Hypotheses 1993 41 542 547 10.1016/0306-9877(93)90111-3 8183132

[B34] Daenke S Bangham CR Do T cells cause HTLV-1-associated disease?: a taxing problem Clin Exp Immunol 1994 96 179 181 8187324 10.1111/j.1365-2249.1994.tb06538.x PMC1534872

[B35] Umehara F Nakamura A Izumo S Kubota R Ijichi S Kashio N Hashimoto K Usuku K Sato E Osame M Apoptosis of T lymphocytes in the spinal cord lesions in HTLV-I-associated myelopathy: a possible mechanism to control viral infection in the central nervous system J Neuropathol Exp Neurol 1994 53 617 624 10.1097/00005072-199411000-00009 7964902

[B36] Umehara F Izumo S Nakagawa M Ronquillo AT Takahashi K Matsumuro K Sato E Osame M Immunocytochemical analysis of the cellular infiltrate in the spinal cord lesions in HTLV-I-associated myelopathy J Neuropathol Exp Neurol 1993 52 424 430 10.1097/00005072-199307000-00010 8355031

[B37] Umehara F Izumo S Ronquillo AT Matsumuro K Sato E Osame M Cytokine expression in the spinal cord lesions in HTLV-I-associated myelopathy J Neuropathol Exp Neurol 1994 53 72 77 10.1097/00005072-199401000-00009 8301322

[B38] Moritoyo T Reinhart TA Moritoyo H Sato E Izumo S Osame M Haase AT Human T-lymphotropic virus type I-associated myelopathy and tax gene expression in CD4+ T lymphocytes Ann Neurol 1996 40 84 90 10.1002/ana.410400114 8687197

[B39] Umehara F Okada Y Fujimoto N Abe M Izumo S Osame M Expression of matrix metalloproteinases and tissue inhibitors of metalloproteinases in HTLV-I-associated myelopathy J Neuropathol Exp Neurol 1998 57 839 849 10.1097/00005072-199809000-00005 9737547

[B40] Copeland KF Heeney JL T helper cell activation and human retroviral pathogenesis Microbiol Rev 1996 60 722 742 8987361 10.1128/mr.60.4.722-742.1996 PMC239461

[B41] Uchiyama T Human T cell leukemia virus type I (HTLV-I) and human diseases Annu Rev Immunol 1997 15 15 37 10.1146/annurev.immunol.15.1.15 9143680

[B42] Itoyama Y Minato S Kira J Goto I Sato H Okochi K Yamamoto N Spontaneous proliferation of peripheral blood lymphocytes increased in patients with HTLV-I-associated myelopathy Neurology 1988 38 1302 1307 10.1212/WNL.38.8.1302 2899862

[B43] Nakamura T Nishiura Y Ichinose K Shirabe S Tsujino A Goto H Furuya T Nagataki S Spontaneous proliferation of and cytokine production by T cells adherent to human endothelial cells in patients with human T-lymphotropic virus type I-associated myelopathy Intern Med 1996 35 195 199 10.2169/internalmedicine.35.195 8785452

[B44] Li Q Verma IM NF-kappaB regulation in the immune system Nat Rev Immunol 2002 2 725 734 10.1038/nri910 12360211

[B45] Vartanian T Li Y Zhao M Stefansson K Interferon-gamma-induced oligodendrocyte cell death: implications for the pathogenesis of multiple sclerosis Mol Med 1995 1 732 743 8612196 PMC2230017

[B46] Yamamoto K Utsunomiya A Tobinai K Tsukasaki K Uike N Uozumi K Yamaguchi K Yamada Y Hanada S Tamura K Phase I study of KW-0761, a defucosylated humanized anti-CCR4 antibody, in relapsed patients with adult T-cell leukemia-lymphoma and peripheral T-cell lymphoma J Clin Oncol 2010 28 1591 1598 10.1200/JCO.2009.25.3575 20177026

[B47] Osame M Review of WHO Kagoshima meeting and diagnostic guidelines for HAM/TSP 1990 New York: Raven Press

[B48] Lee B Tanaka Y Tozawa H Monoclonal antibody defining tax protein of human T-cell leukemia virus type-I Tohoku J Exp Med 1989 157 1 11 10.1620/tjem.157.1 2711372

[B49] Saito M Matsuzaki T Satou Y Yasunaga J Saito K Arimura K Matsuoka M Ohara Y In vivo expression of the HBZ gene of HTLV-1 correlates with proviral load, inflammatory markers and disease severity in HTLV-1 associated myelopathy/tropical spastic paraparesis (HAM/TSP) Retrovirology 2009 6 19 10.1186/1742-4690-6-19 19228429 PMC2653460

