# Supplementary material for: Increased expression of OX40 is associated with progressive disease in patients with HTLV-1-associated myelopathy/tropical spastic paraparesis
Source: Retrovirology. 2013 May 7;10:51. doi: 10.1186/1742-4690-10-51 (PMC3659064; doi:10.1186/1742-4690-10-51)
Supplement: Additional file 1: Figure S1 — OX40 was expressed on the surface of Tax+ CD4+ T cells from HTLV-1 infected individuals. OX40 was detected on CD4+ T cells of HAM/TSP patients (HAM/TSP3, 4) and AC (AC1) with anti-OX40 mAb (clones B-7B5) after 16 hours in vitro cultivation in the absence of any growth factors or mitogen (center panels). OX40 was expressed almost exclusively in naturally infected CD4+ T cells that also expressed Tax (right panels). FigureS2. The expression of 4-1BB on CD4+ T cells from HAM/TSP patients. A. 4-1BB was detected on both CD4+ and CD4- T cells of HAM/TSP patients with anti-4-1BB mAb (clone 4B4, eBioscience) after 16 hours in vitro cultivation in the absence of any growth factors or mitogen. B. Tax protein was detected in CD4+ T cells after 16 hours in vitro cultivation. C. The expression of 4-1BB was associated with the expression of Tax. Figure S3. Functional OX40 is specifically expressed on the surface of T cells naturally infected with HTLV-1. To determine if cell surface OX40 is functional, flow cytometry based binding assays have been carried out. Aliquots of Fc-blocked cells were incubated with biotinylated recombinant soluble OX40L at a concentration of 2.5 mg/ml for 30 min on ice. Then cells were washed and stained with PE-streptavidin (Biolegend) and PC5-labeled anti-CD4 for 30 min on ice. After washing, the cells were fixed and processed to detect concomitantly Tax (see Methods). The frequency of CD4+ T cells that were positively stained with biotinylated recombinant soluble OX40L and PE-streptavidin was similar to the percentage of CD4+ T cells stained by anti-OX40 mAb, indicating that these cells expressed functional OX40. [file 1742-4690-10-51-S1.ppt]

## Slide 1
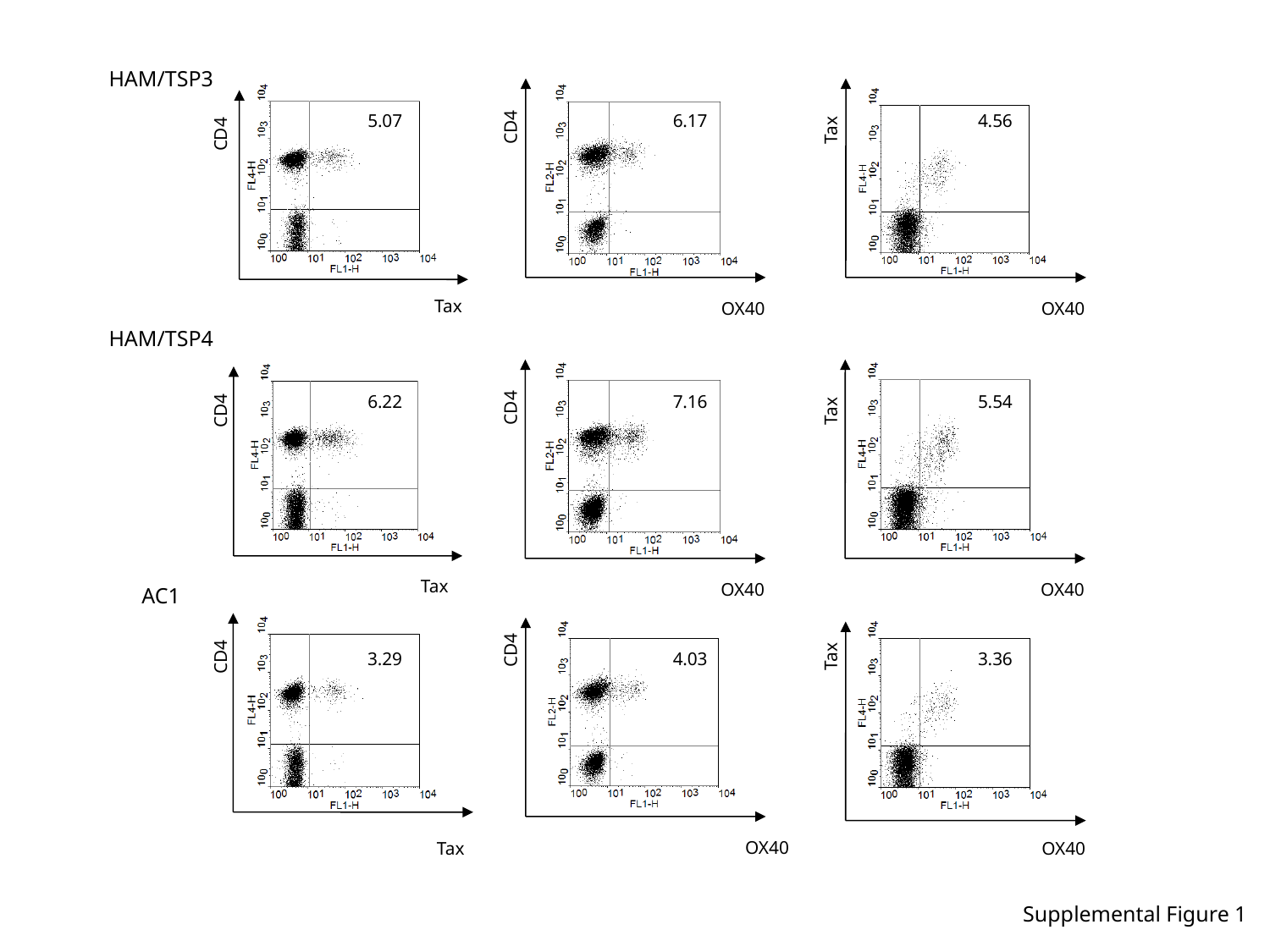

HAM/TSP3
5.07
6.17
4.56
CD4
Tax
CD4
Tax
OX40
OX40
HAM/TSP4
6.22
7.16
5.54
CD4
CD4
Tax
Tax
OX40
OX40
AC1
CD4
Tax
CD4
3.29
4.03
3.36
OX40
Tax
OX40
Supplemental Figure 1

## Slide 2
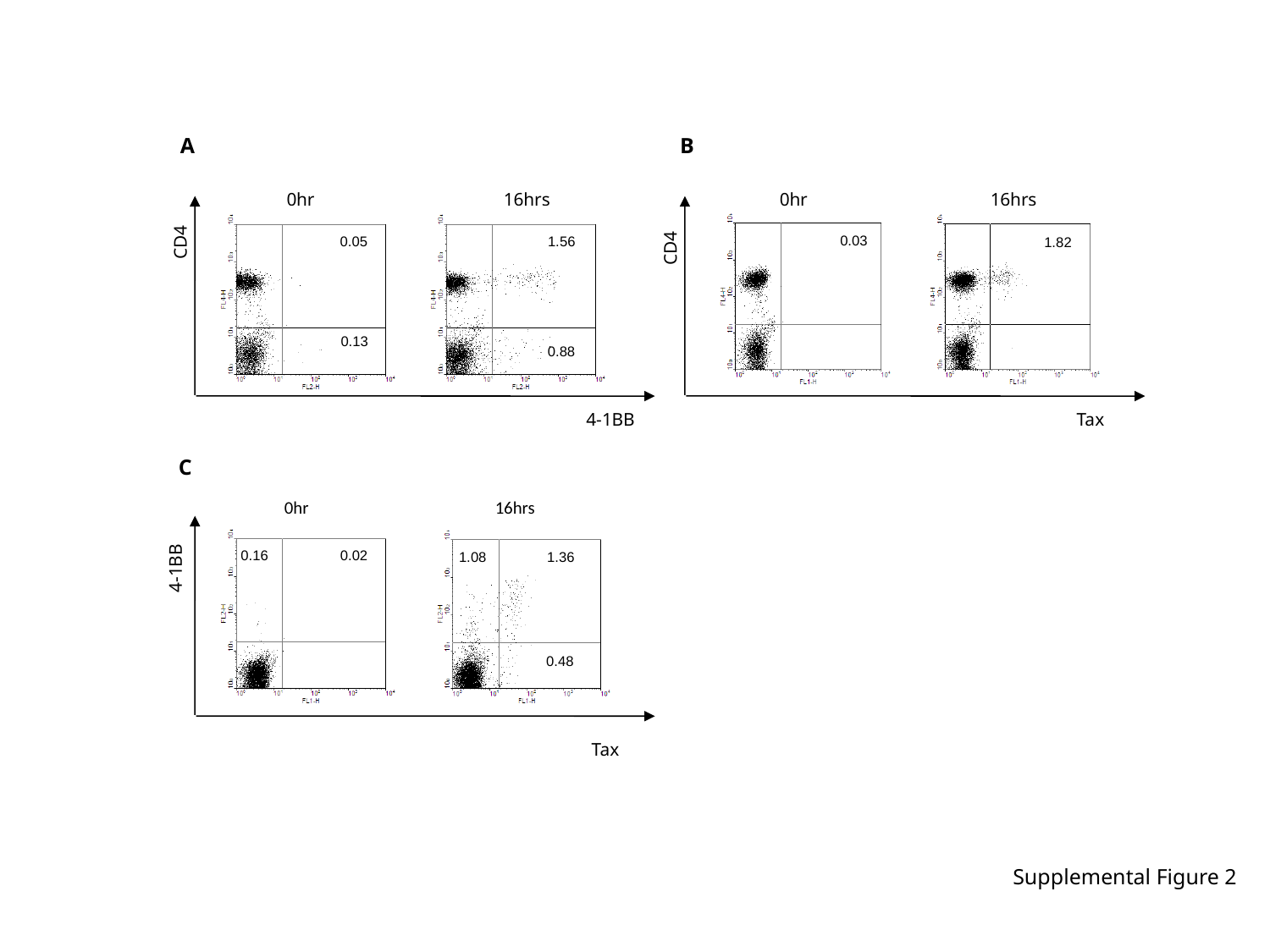

B
A
0hr
16hrs
0hr
16hrs
CD4
0.03
0.05
1.56
1.82
CD4
0.13
0.88
4-1BB
Tax
C
0hr
16hrs
0.16
0.02
1.08
1.36
4-1BB
0.48
Tax
Supplemental Figure 2

## Slide 3
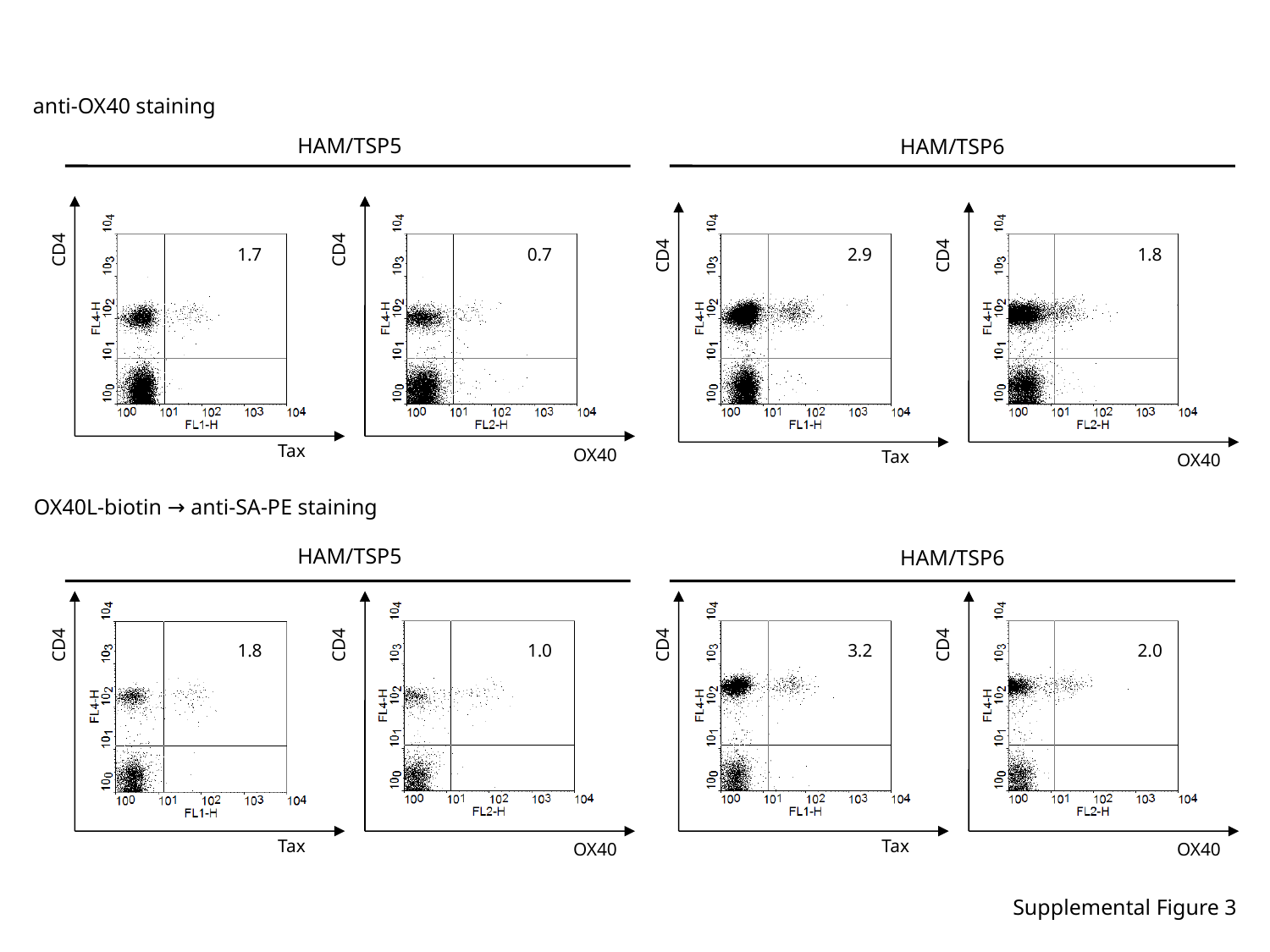

anti-OX40 staining
HAM/TSP5
HAM/TSP6
CD4
CD4
1.7
0.7
2.9
1.8
CD4
CD4
Tax
OX40
Tax
OX40
OX40L-biotin → anti-SA-PE staining
HAM/TSP5
HAM/TSP6
CD4
CD4
CD4
CD4
1.8
1.0
3.2
2.0
Tax
Tax
OX40
OX40
Supplemental Figure 3
