# Supplementary material for: Increased expression of OX40 is associated with progressive disease in patients with HTLV-1-associated myelopathy/tropical spastic paraparesis
Source: Retrovirology. 2013 May 7;10:51. doi: 10.1186/1742-4690-10-51 (PMC3659064; doi:10.1186/1742-4690-10-51)
Supplement: Additional file 2: Table S1 — Ex vivo frequency of OX40 and Tax positive T cells in peripheral blood mononuclear cells from HTLV-1 infected individuals. [file 1742-4690-10-51-S2.doc]

**Supplemental Table 1**

**Ex vivo frequency of OX40 and Tax positive T cells in peripheral blood mononuclear cells from HTLV-1 infected individuals.**

| Case | Age | Sex | %OX40+CD4^+a^ | %Tax+CD4^+^ | %Tax+ OX40^+^ | Proviral load^b^ |
| --- | --- | --- | --- | --- | --- | --- |
| HAM/TSP1 | 74 | F | 3.62 | 5.09 | 3.67 | 687 |
| HAM/TSP2 | 42 | M | 2.90 | 1.83 | 1.73 | 800 |
| HAM/TSP3 | 39 | F | 6.17 | 5.07 | 4.56 | 1695 |
| HAM/TSP4 | 82 | F | 7.16 | 6.22 | 5.54 | 630 |
| mean±SE | 59.3±11.0 |  | 4.96±1.01 | 4.55±0.95 | 3.88±0.81 | 953.0±249.8 |
| AC1 | 72 | M | 4.03 | 3.29 | 3.36 | 317 |
| AC2 | 29 | F | 0.68 | 0.96 | 0.53 | 69 |
| AC3 | 48 | F | 0.40 | 0.53 | 0.31 | 187 |
| mean±SE | 49.7±12.4 |  | 1.70±1.17 | 1.59±0.86 | 1.40±0.98 | 191.0±71.6 |

HAM/TSP: HTLV-1 associated myelopathy/tropical spastic paraparesis AC: asymptomatic carrier

^a^ %OX40^+^CD4^+^ means the frequency of OX40^+^CD4^+^ in peripheral blood mononuclear cells (PBMCs)

^b^ Proviral load: HTLV-1 tax copy number per 10^4^ PBMCs
